# Gene expression profile of bladder tissue of patients with ulcerative interstitial cystitis

**DOI:** 10.1186/1471-2164-10-199

**Published:** 2009-04-28

**Authors:** Marianne Gamper, Volker Viereck, Verena Geissbühler, Jakob Eberhard, Jochen Binder, Carlo Moll, Hubert Rehrauer, René Moser

**Affiliations:** 1 Biomedical Research Foundation, Lauchefeld 31, 9548 Matzingen, Switzerland; 2 Department of Gynecology and Obstetrics, Kantonsspital Frauenfeld, 8501 Frauenfeld, Switzerland; 3 Department of Urology, Kantonsspital Frauenfeld, 8501 Frauenfeld, Switzerland; 4 Institute for Pathology, Spital Thurgau AG, 8596 Scherzingen, Switzerland; 5 Functional Genomics Center Zurich, ETH Zurich and University of Zurich, 8057 Zurich, Switzerland; 6 IBR Inc, Institute for Biopharmaceutical Research, Lauchefeld 31, 9548 Matzingen, Switzerland

## Abstract

**Background:**

Interstitial cystitis (IC), a chronic bladder disease with an increasing incidence, is diagnosed using subjective symptoms in combination with cystoscopic and histological evidence. By cystoscopic examination, IC can be classified into an ulcerative and a non-ulcerative subtype. To better understand this debilitating disease on a molecular level, a comparative gene expression profile of bladder biopsies from patients with ulcerative IC and control patients has been performed.

**Results:**

Gene expression profiles from bladder biopsies of five patients with ulcerative IC and six control patients were generated using Affymetrix GeneChip expression arrays (Affymetrix – GeneChip^® ^Human Genome U133 Plus 2.0). More than 31,000 of > 54,000 tested probe sets were present (detection p-value < 0.05). The difference between the two groups was significant for over 3,500 signals (t-test p-value < 0.01), and approximately 2,000 of the signals (corresponding to approximately 1,000 genes) showed an IC-to-healthy expression ratio greater than two. The IC pattern had similarities to patterns from immune system, lymphatic, and autoimmune diseases. The dominant biological processes were the immune and inflammatory responses. Many of the up-regulated genes were expressed in leukocytes, suggesting that leukocyte invasion into the bladder wall is a dominant feature of ulcerative IC. Histopathological data supported these findings.

**Conclusion:**

GeneChip expression arrays present a global picture of ulcerative IC and provide us with a series of marker genes characteristic for this subtype of the disease. Evaluation of biopsies from other bladder patients with similar symptoms (e.g. patients with non-ulcerative IC) will further indicate whether the data presented here will be valuable for the diagnosis of IC.

## Background

Interstitial cystitis (IC) is a chronic bladder disorder characterized by a combination of symptoms including pelvic pain, urinary urgency, and urinary frequency. The estimated prevalence of IC ranges from 18 to over 400 cases per 100,000 individuals, with a predominance of cases found in women [1-3]. Upon cystoscopy, IC can be divided into a classic disease with Hunner's ulcer and a non-ulcerative disease [4-6]. The relationship between these two subtypes is not known and it has been suggested that they represent separate disease entities [5]. Patients with classic IC are older and have a lower bladder capacity than patients with non-ulcerative IC [5,6]. Histological examination of bladder biopsies from classic IC patients reveals mucosal ulceration and extensive inflammation [4,7]. The etiology of IC is unknown, but many theories have been proposed, including neurogenic inflammation with mast cell activation; absorption of potentially toxic substances across the urothelium; a deficient glycosaminoglycan mucus layer and a "leaky" urothelium triggering a chronic inflammatory reaction in the subepithelial layers; or an allergic or autoimmune process [1,3]. There is no evidence that IC is caused by bacterial infection [1].

Because no molecular marker assay is available, IC today is diagnosed by symptom assessment, physical examination, routine urine analysis, cystoscopy, and sometimes bladder biopsy. The National Institute of Diabetes and Digestive and Kidney Diseases (NIDDK) elaborated a set of inclusion and exclusion criteria for the diagnosis of IC for research purposes [2]. Inclusion criteria require a cystoscopy under anesthesia positively identifying Hunner's ulcer or glomerulations (bleedings of the bladder wall upon hydrodistension). Other inclusion criteria are bladder pain and/or urinary urgency [2]. The NIDDK excludes patients with a bladder capacity larger than 350 ml, patients with a symptom duration of less than nine months, patients with urinary frequency less than eight times a day and with an absence of nocturia, and patients who can successfully be treated with antimicrobials, urinary antiseptics, anticholinergics or antispasmodics. The NIDDK criteria can be fully satisfied by 40% of all IC patients [8].

Mast cells may play a central role in the pathogenesis and pathophysiology of IC [9-11]. Mast cells migrate into tissue perivascular spaces, and upon activation secrete granule-stored or de novo-synthesized molecules that mediate inflammatory reactions [9].

Research has focused on finding objective markers for this disease. Urine analysis of IC patients who fulfilled the NIDDK criteria showed an increase in antiproliferative factor (APF), a decrease in heparin-binding epidermal growth factor-like growth factor (HB-EGF), and an increase in epidermal growth factor (EGF) [12]. APF, a glycosylated nonapeptide [13], is the biomarker with the most specificity for IC [14], suggesting its potential use in diagnostic testing. However, no test for APF is commercially available, and the regulation of APF production, as well as APF's role in causing symptoms, are uncertain [3]. Many APF experiments were done with explanted epithelial cells in culture [15] – a condition that only represents one aspect of the in vivo situation. More potential IC markers have been identified by urine analysis. These are, for example, inter-alpha-trypsin inhibitor heavy chain H4 [16], neutrophil elastase [17], insulin-like growth factor binding protein-3 [14], IL-6 [14] or glycoprotein GP51 [18].

Directly analyzing gene expression in bladder biopsies allows us to use a different approach for the molecular characterization of IC. We looked for any significant differences in mRNA levels between bladder cells from IC patients with ulcers vs. bladder cells from healthy controls. Even though ulcerative IC represents only a fraction of all IC patients, this criteria allows for examination of a relatively homogenous patient population. The global approach of gene expression arrays has offered new information on ulcerative IC and has provided us with data for defining a characteristic pattern of "marker genes" for this subtype of the disease.

## Results

### Description of patients

To accurately define a characteristic pattern of "marker genes", patient selection was crucial. Creating a relatively homogenous patient population required including only IC patients with Hunner's ulcer. Table 1 summarizes the clinical characteristics of the five IC and the six control patients selected for our study. All patients are white females, and for all patients an acute bacterial infection was excluded. In urine specimens collected prior to surgery, no bacterial growth was found. Only one patient, classic IC patient 15, had leukocytes in the urine sample. Since this patient has very severe glomerulations, this was due to acute bleeding rather than due to a urinary tract infection. There also was no evidence for a chronic urinary tract infection since PCR analyses of representative bladder biopsies were negative for a selection of bacterial or viral pathogens (results not shown). Four control patients did not have any symptoms related to IC, but underwent vaginal prolapse surgery after biopsy removal. Two control patients, patient 2 and patient 10, had bladder-related symptoms. Patient 2 had urgency and frequency for 20 months, only minor pain, and a bladder capacity of 400 ml. Thus, this patient meets the relaxed eligibility criteria for the IC Database Study [19]. Histopathology data analysis from patient 2 shows an intact urothelium with a slight increase in mast cell counts in the detrusor muscle, suggesting a mild form of IC. Patient 10 did not meet the inclusion criteria for the IC Database Study, having neither urgency nor frequency and a localized pain sensation for only three months. Upon histopathological analysis, patient 10 was diagnosed with Cystitis glandularis et cystica. IC patients with ulcers are relatively old and the age difference between the two investigated patient groups was close to statistical significance (p-value 0.052; Table 1). All patients except one (control patient 2) are postmenopausal. Previous studies also report an older age for ulcerative patient groups [5-7]. No common medical history was found among the patients. Of note is that one of the classic IC patients has chronic polyarthritis, and another has polymyalgia rheumatica, but none of the patients have a history of a lymphatic disease.

**Table 1:** Clinical characteristics of five IC patients and six control patients

		**IC patients**	**control group**	**p-value**	**sig.**
Age [year]	Mean	78.0	59.2	0.052*	ns
	Standard dev.	7.5	13.2		
	Median	82.0	57.5		
	Minimum	68.0	40.0		
	Maximum	85.0	75.0		

Duration of symptoms [month]	Mean	32.6	7.7	0.143*	ns
	Standard dev.	23.6	10.8		
	Median	24.0	3.0		
	Minimum	13.0	0.0		
	Maximum	72.0	20.0		

Frequency [/24 h]	Mean	14	8	0.056*	ns
	Standard dev.	9	2		
	Median	10	7		
	Minimum	8	7		
	Maximum	30	11		

Average volume [< 350 ml]		5/5	0/6	0.002^§^	s

Nocturia	Mean	4	1	0.032*	s
	Standard dev.	2	1		
	Median	3	1		
	Minimum	2	0		
	Maximum	8	2		

Pain, VAS [0–100]	Mean	69.4	19.0	0.016*	s
	Standard dev.	9.4	26.0		
	Median	73.0	10.5		
	Minimum	55.0	0.0		
	Maximum	78.0	55.0		

Urge, VAS [0–100]	Mean	69.6	32.0	0.111*	ns
	Standard dev.	19.0	38.2		
	Median	76.0	26.0		
	Minimum	37.0	0.0		
	Maximum	83.0	76.0		

Hunner's ulcer		5/5	0/6	0.002^§^	s

Glomerulations		4/5	0/6	0.015^§^	s

Bladder tumor		0/5	0/6	n.a.	

Diverticulum		0/5	0/6	n.a.	

* Mann-Whitney-test, significance level = 0.050

^§ ^Chi-Square-test, significance level = 0.050

n.a.: not applicable

VAS: visual analogue scale

### Raw data evaluation

**Figure 1:**
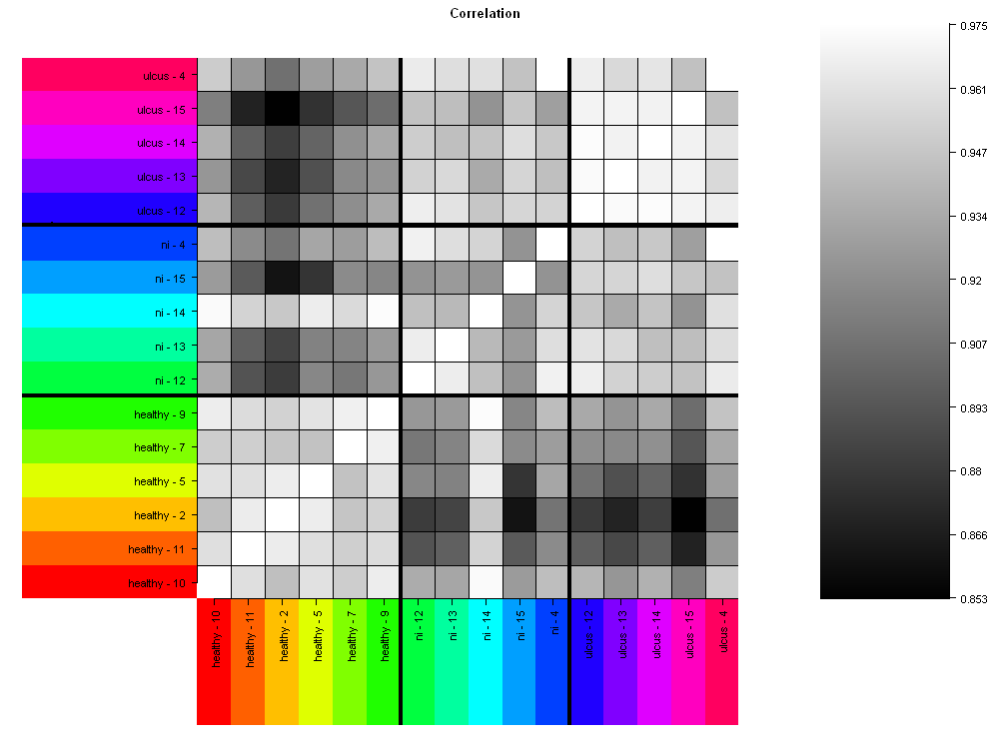
**Gene expression array correlation plot**. Gene expressions of 16 bladder biopsies were compared. Bladder tissue was taken from six healthy controls ("healthy") and from five IC patients ("ulcus" and "ni"). From each IC patient, two samples have been analyzed, one from an ulcer (ulcus) and one from a non-ulcer (ni) area. Only probe sets defined as "present" have been included in this comparison, and the metric was the Pearson correlation coefficient. White or light-grey squares represent a similar gene expression, and black or dark-grey squares represent a difference in gene expression. Ulcer tissue (ulcus); non-ulcer tissue in patients with Hunner's ulcers (ni).

From each IC patient, two bladder biopsies were analyzed, one from an ulcer area ("ulcus"), and one from a non-ulcer area ("ni"). The correlation plot shows the pair-wise comparison of all chips (Figure 1). White or light grey squares correspond to similar gene expression, and black or dark-grey squares correspond to different gene expression. Gene expression among the healthy control group and also among the ulcerative group shows a good correlation (light squares). A comparison of all IC patient samples (ulcus and ni) with the healthy controls reveals a clear difference in gene expression (dark squares). One exception is patient sample 14 ni that has a gene expression similar to the healthy controls. Gene expression of sample 15 ni is different from the other four ni samples: 4 ni, 12 ni, 13 ni and 14 ni. There are only minor differences between ulcer and ni samples. Previous research has primarily focused on areas of the bladder with the most severe pathology [7,20,21]. However, the results from the correlation plot (Figure 1) encouraged us to perform the statistical evaluations (t-test) for the five ni samples (4 ni, 12 ni, 13 ni, 14 ni, 15 ni) in comparison to the six healthy controls (2, 5, 7, 9, 10, 11).

### GeneChip expression data analysis

**Figure 2:**
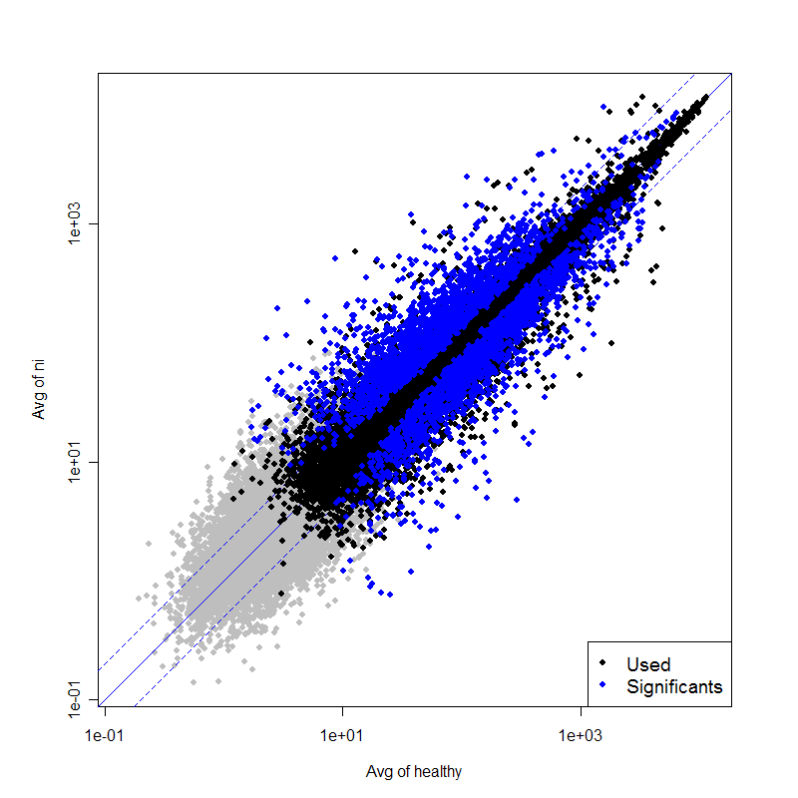
**Scatter plot: ni-vs-healthy**. The scatter plot illustrates the data selection set by the statistical requirements in the ni-vs-healthy comparison. The average expression values of condition "healthy" (patients 2, 5, 7, 9, 10, 11; x-axis) and condition "ni" (patients 4, 12, 13, 14, 15; y-axis) were compared. For this graph, all normalized data (54,613 probe sets) were used. Grey dots correspond to "not present" probe sets (criteria for "present": detection p-value < 0.05). Black dots correspond to "presents", and blue dots correspond to "presents" and "significants" (criteria for "significant": p-value for differential expression < 0.01). Non-ulcer tissue in patients with Hunner's ulcers (ni).

GeneChip expression arrays were used to compare the population of ulcerative IC patients (five ni samples) with the population of healthy controls (six control samples). From a total of 54,613 tested probe sets 31,579 (58%) were found to be present (detection p-value < 0.05) (see Additional file 1). The difference in mRNA levels between the two groups is significant for 3,618 probe sets as determined by the t-test statistics (p-value < 0.01; false discovery rate (FDR) 8.7%) (see Additional file 2). Using these statistical constraints, we were able to focus on genes expressed in the mid-to-high range (Figure 2). Of the 3,618 significant probe sets, 1,957 showed an IC (ni) to healthy expression ratio greater than two (see Additional file 3). This corresponds to 1,417 proteins that are potentially over- or under-represented in ulcerative IC by a factor of two or more (see Additional file 4).

**Figure 3:**
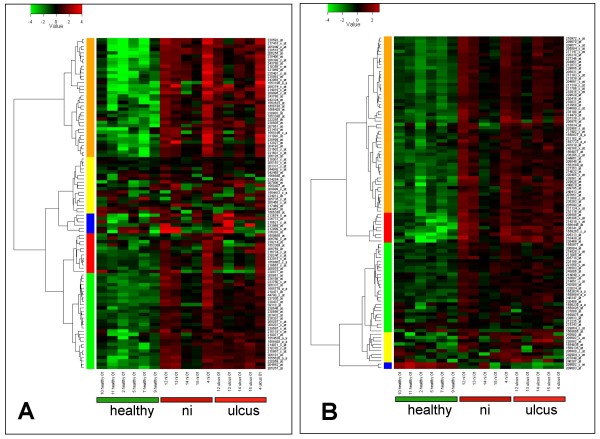
**Heatmap visualization of the top 100 B- and T-cell markers**. Using a biomarker search with Genevestigator [33] we identified the top 100 probe sets that were specific for "B-lymphocytes" (A) and "T-lymphocytes" (B), respectively, and visualized them as a heatmap. For each probe set the heatmap shows the log2-ratio against the average of our entire data set. For both the B- and T-lymphocytes, a large proportion of the probe sets was upregulated in "ni" and "ulcus" as compared to the healthy tissue. Additional file 6 (Blympho-top100 cluster) and Additional file 7 (Tlympho-top100 cluster) list the top 100 probe sets and their corresponding gene names. Ulcer tissue (ulcus); non-ulcer tissue in patients with Hunner's ulcers (ni).

Interestingly, many of the 25 proteins with the highest IC-to-healthy expression ratios are expressed in B-leukocytes (Table 2, [22-31], see Additional file 5). Therefore, B-cell invasion into the bladder wall could be proposed as a dominant feature of ulcerative IC. Our results also show that characteristic genes for T-lymphocytes are significantly up-regulated in ulcerative IC; however, they do not rank among the top 25. As urothelial cells can also be involved in immune response [32], the possibility that gene expressions in the urothelium make a minor contribution to these findings cannot be excluded. However, the urothelial cells represent only a small fraction of our biopsy samples (see below). Strong evidence for B- and T-cell infiltration came from cluster analysis using Genevestigator [33]. This reference expression database and meta-analysis system predicted the top 100 markers for both B- and T-lymphocytes, respectively. The two lists of probe sets were generated from the database independent of our results (see Additional file 6 and Additional file 7). Interestingly, many top markers are significantly up-regulated in our ulcerative IC patient samples (Figures 3A, B) and convincingly confirm B- and T-cell infiltration of the bladder wall. Nine of the top 25 gene expressions (IGHM, MS4A1, FCRL3, IGHG1, PAX5, BLK, IGHA1, IGL@, FCRLA; Table 2) were present in the top 100 probe sets for B-lymphocytes (see Additional file 6).

**Table 2:** Top 25 gene expressions in ulcerative IC

**#**	gene symbol*	**protein**	**protein name**	**over-expression**$
1	CHI3L1	CH3L1_HUMAN	Chitinase-3-like protein 1	68
2	CXCL13	CXL13_HUMAN	C-X-C motif chemokine 13	59
3	**CR2, CD21 **[22]	CR2_HUMAN	Complement receptor type 2	47
4	LTF	TRFL_HUMAN	Lactotransferrin	44
5	**IGHM **[23]	IGHM_HUMAN	Ig mu chain C region	37
6	**MS4A1 **[24]	CD20_HUMAN	B-lymphocyte antigen CD20	32
7	**IGHG1 **[25]	IGHG1_HUMAN	Ig gamma-1 chain C region	26
8	**IGL@ **[26]	IGL@	immunoglobulin lambda locus	24
9	CCL18	CCL18_HUMAN	C-C motif chemokine 18	23
10	**FCRLA **[27]	FCRLA_HUMAN	Fc receptor-like A	22
11	**IGH@ **[25]	IGH@	immunoglobulin heavy locus	21
12	MGC29506	PACAP_HUMAN	Proapoptotic caspase adapter protein	20
13	**FCRL5 **[28]	FCRL5_HUMAN	Fc receptor-like protein 5	20
14	**IGHA1 **[25]	IGHA1_HUMAN	Ig alpha-1 chain C region	19
15	**PAX5 **[29]	PAX5_HUMAN	Paired box protein Pax-5	19
16	**FCRL3 **[28]	FCRL3_HUMAN	Fc receptor-like protein 3	19
17	**BLK **[30]	BLK_HUMAN	Tyrosine-protein kinase BLK	19
18	NTN2L	NET2_HUMAN	Netrin-2-like protein	18
19	TDO2	T23O_HUMAN	Tryptophan 2,3-dioxygenase	18
20	CLC	LPPL_HUMAN	Eosinophil lysophospholipase	16
21	**IGHV1–69 **[31]	IGHV1–69	immunoglobulin heavy variable 1–69	16
22	RPL14	RL14_HUMAN	60S ribosomal protein L14	16
23	**FCRL1 **[28]	FCRL1_HUMAN	Fc receptor-like protein 1	16
24	AQP9	AQP9_HUMAN	Aquaporin-9	15
25	IL8	IL8_HUMAN	Interleukin-8	15

* Gene expressions characteristic for B-cells are typed in **BOLD**.

$ Data from Additional file 4; for more information see Additional file 5.

### Histopathology data

**Figure 4:**
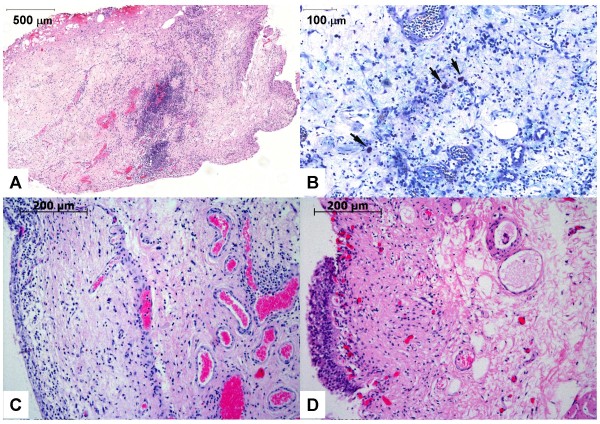
**Histopathology data**. Representative histopathology findings of bladder biopsies from IC patient 15 (A, B), from IC patient 4 (C), and from control patient 2 (D). (A) Cross-section of an entire biopsy (60× magnification). The HE stain shows a focally accentuated lymphoplasmocytic inflammatory infiltration. The inner bladder surface is denudated from the urothelial lining (lower right), thus showing an ulcer area. (B) Giemsa staining (200×). Arrowheads point to numerous mast cells in the mixed lymphoplasmocytic inflammatory infiltration. Blood vessels show active hyperemia with dilated lumina. There is evidence of a substantial interstitial edema. (C) The HE stain (100×) shows a marginal urothelial layer (on the left) and substantial inflammation in deep areas of the biopsy. (D) The HE stain (100×) shows the urothelial layer on the left side and signs of a mild unspecific subepithelial inflammation.

Biopsies of IC patients and control patients were also investigated microscopically. Ideally, three layers of the bladder wall, namely the urothelium (mucosa), the submucosa (lamina propria) and the muscularis propria (detrusor muscle), are included in a single biopsy. Histological data of the biopsies from our IC patients showed a complete (Figure 4A) or a partial (Figure 4C) denudation of the urothelium, a feature commonly found in classic IC [4,7,20]. Care was taken to remove biopsies of similar sizes; however, the depths of the biopsies vary, as do the relative ratios of the cell layers. For all IC patients, hematoxylin and eosin (HE) staining of representative bladder sections confirmed a severe inflammation characterized by lymphocyte and plasma cell invasion in the deeper areas (muscularis propria) of the biopsies (Figures 4A, C). These findings strongly support our gene expression data and agree with previously reported results [4,7,20,21,34]. The submucosa of control patient 2 was covered by a urothelial layer (Figure 4D). Even though the control showed a minor unspecific subepithelial inflammation (Figure 4D), deeper areas of the biopsy did not demonstrate the severe leukocyte infiltration as found in ulcerative IC (Figure 4C). Mastocytosis has been credited with a central role in the pathogenesis and pathophysiology of IC [9-11]. All of our classic IC patients showed an increase in mast cells in the detrusor muscle and in the lamina propria (Figure 4B).

### Important processes in ulcerative IC

The ermineJ software program identified gene ontology (GO) categories that are overrepresented in the significant genes found for ulcerative IC [35]. We examined overrepresentation of GO categories in the up- and down-regulated genes separately. The significance of overrepresentation of GO categories is much higher for up-regulated genes than for down-regulated genes (Table 3). In the three ontologies "biological process," "molecular function," and "cellular component," the best overlaps were found for the GO categories "immune system process" (corrected p-value 1.88E-74), "signal transducer activity" (corrected p-value 1.17E-22), and "plasma membrane" (corrected p-value 1.26E-15), respectively (Table 3). These data support our findings of leukocyte infiltration in ulcerative IC. They furthermore suggest that chemotactic signals are recognized by receptors in the plasma membrane of these cells, which in turn elicit an intracellular signaling cascade eventually regulating gene expression.

**Table 3:** Most significant GO categories in ulcerative IC

**result file**	**term**	**ID**	**count**	**size**	**p-value**	corrected p-value*
biological processes up	immune system process	GO:0002376	217	551	1.07E-77	1.88E-74
	immune response	GO:0006955	180	418	4.21E-71	7.41E-68
	response to stimulus	GO:0050896	308	1397	1.36E-44	2.39E-41
	defense response	GO:0006952	117	325	2.19E-36	3.84E-33
	signal transduction	GO:0007165	348	2177	1.36E-20	2.39E-17
	cell communication	GO:0007154	371	2387	5.63E-20	9.89E-17
	inflammatory response	GO:0006954	66	195	3.71E-19	6.51E-16

biological processes down	transmembrane receptor protein serine/threonine kinase signaling pathway	GO:0007178	18	55	1.99E-07	3E-04

molecular functions up	signal transducer activity	GO:0004871	234	1200	1.48E-25	1.17E-22
	receptor activity	GO:0004872	170	854	3.00E-19	2.35E-16

molecular functions down	sequence-specific DNA binding	GO:0043565	46	293	1.32E-05	0.0104

cellular components up	plasma membrane	GO:0005886	228	1304	3.27E-18	1.26E-15

cellular components down	none	-	-	-	-	-

* Up-regulated GO categories with a corrected p-value < 1.3E-15 are listed, the top-down regulated GO category of the respective analysis is shown. For "cellular components down" no significant GO category has been found.

In MetaCore analysis, immune reaction processes were found to be dominant in ulcerative IC (Table 4). The top process found was "Cell adhesion_Leucocyte chemotaxis". The six cytokine/chemokine genes encoding CXCL13, CCL18, IL-8, CCL19, CXCL6 and GROa rank within the top seven genes of this process. Receptors and adhesion molecules are also over-represented (see Additional file 8).

**Table 4:** The 20 most significant affected processes in ulcerative IC

**#**	**name**	**p-value**	**ratio**
1	Cell adhesion_Leucocyte chemotaxis	3.05E-18	78/206
2	Immune_Phagosome in antigen presentation	1.43E-14	81/248
3	Immune_TCR signaling	2.55E-14	59/154
4	Immune_Antigen presentation	3.33E-14	68/193
5	Immune_Phagocytosis	1.88E-13	72/217
6	Immune_BCR pathway	3.48E-13	53/137
7	Inflammation_Neutrophil activation	2.12E-12	71/222
8	Inflammation_NK cell cytotoxicity	2.66E-11	54/155
9	Inflammation_TREM1 signaling	6.44E-11	51/145
10	Chemotaxis	5.47E-10	47/135
11	Inflammation_Interferon signaling	3.21E-08	38/110
12	Inflammation_Histamine signaling	4.01E-08	60/215
13	Proliferation_Lymphocyte proliferation	2.52E-07	50/175
14	Inflammation_Amphoterin signaling	2.63E-07	38/118
15	Cytoskeleton_Regulation of cytoskeleton rearrangement	6.46E-07	51/185
16	Inflammation_IL-2 signaling	2.33E-06	33/104
17	Cell adhesion_Platelet aggregation	4.76E-06	46/171
18	Inflammation_IL-4 signaling	9.46E-06	34/115
19	Inflammation_Innate inflammatory response	1.76E-05	46/179
20	Inflammation_IgE signaling	1.79E-05	38/138

The two processes "BCR pathway" (B-cell antigen receptor-pathway) and "TCR signaling" (T-cell antigen receptor signaling) were found to be among the most significant processes (Table 4). In TCR signaling, two signals of antigen-presenting-cells are necessary to stimulate T-cells. These are CD80/CD86 and class II MHC on the antigen presenting cell, and CD28 and TCR on the T-cell [36]. The genes encoding these proteins are overexpressed in our array data.

**Figure 5:**
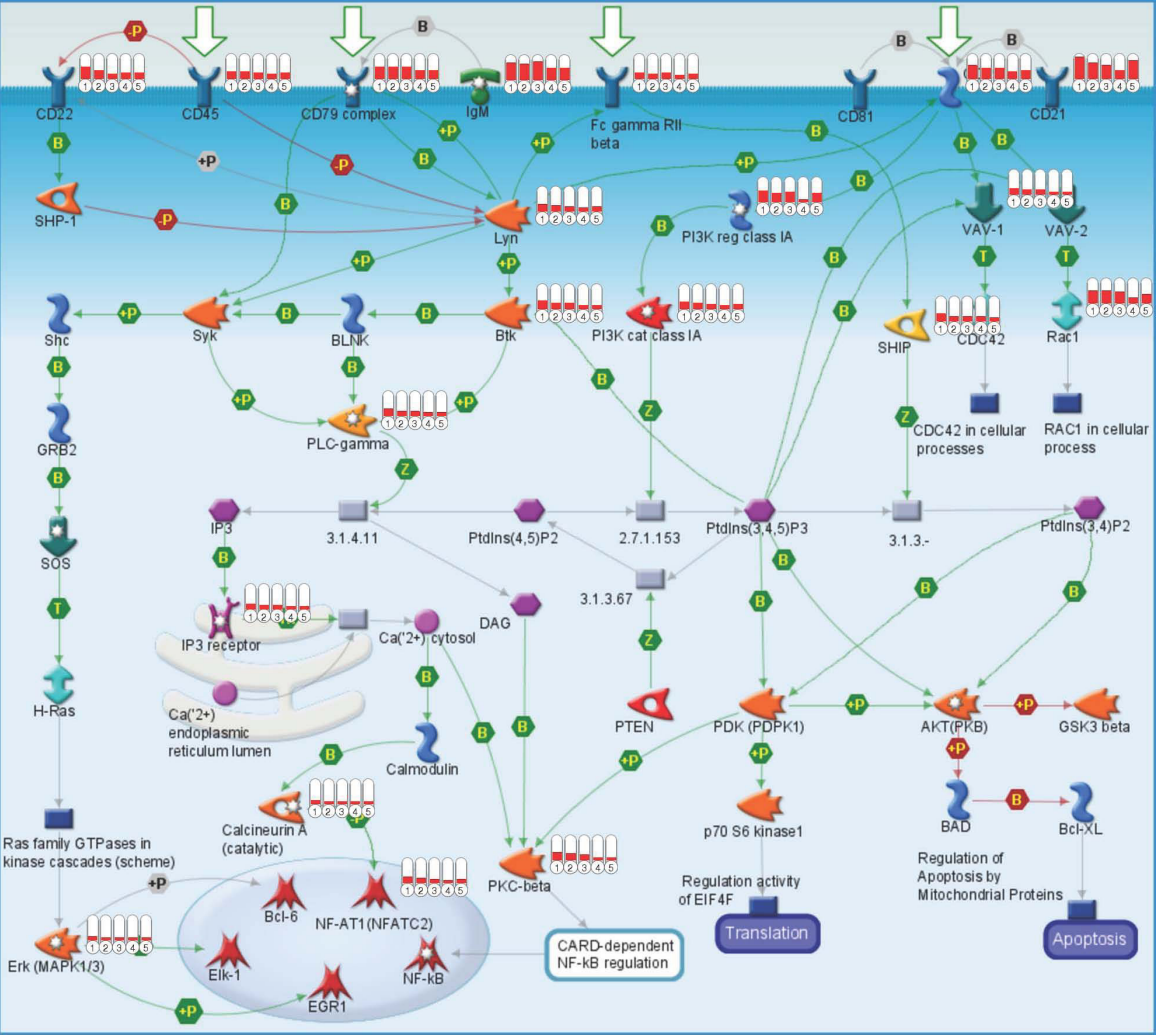
**Map of BCR-pathway**. The process "Immune_BCR pathway" turned out to be significant (p-value 3.48E-13) in ulcerative IC (MetaCore analysis). The map shows a B-cell with the signal transduction from the membrane receptors to the transcription factors in the nucleus. The five columns on the right of an individual signaling component indicate the expression results of the five patient samples (4 ni, 12 ni, 13 ni, 14 ni, 15 ni) relative to the healthy controls. Up-regulated gene expressions are shown in red.

In Figure 5, the map of the BCR pathway is shown with the log ratios of the ni-samples over the healthy samples, visualized as thermometers. Only pathway components with red thermometers, indicating up-regulation in the ni-samples, were found. The B-cell antigen receptor pathway is involved in the development, survival and activation of B lymphocytes [37]. Membrane immunoglobulins bind to their specific antigens, and together with the CD79A/CD79B heterodimer, transduce the signal via a cytoplasmic signaling cascade involving protein tyrosine kinases Lyn and Btk and phospholipase γ (PLC-gamma), activating transcription factors NF-AT or NF-κ B. These factors in turn play key roles in the induction of immune response genes and inflammation [38]. A key regulatory element in B-cell development is transcription factor PAX5 (19× overexpressed; Table 2). Gene expression profile studies in mouse models suggest that PAX5 is essential for B-cell commitment [39,40]. PAX5 promotes B lymphopoiesis by activating B-cell specific genes (among others *Cd19 *and *Cd79a*); is essential for *V-DJ *recombination of the *Igh *locus; maintains B-cell fate in more mature cells; and represses genes whose expression is not B-cell specific. Even though these studies cannot not be directly compared to our experiments, it is interesting to note that the PAX5-dependent genes *Cd19 *and *Cd79a *were among the highly expressed genes found in our study (12× and 14× overexpressed, respectively; see Additional file 4).

### Comparison of the gene expression profiles of ulcerative IC and other diseases

The GeneGo software program defines signature genes for diseases by collecting all available data related to a certain disease (e.g. mRNA expression, proteomics, metabolomics, microRNA assays and other phenotypic data) [41]. By using this computational tool, overlaps of our significant gene expression data with the signature genes of other diseases can be identified, and the significance of these overlaps can be determined.

The forty disease categories having the most similar gene expression to that found in bladder biopsies of ulcerative IC patients are summarized in Table 5. Immune system diseases, lymphatic diseases and autoimmune diseases are the most significant. These categories include different numbers of proteins. Large categories, such as "immune system diseases" (2,482 proteins, 582 of them significantly regulated in ulcerative IC) are more general and contain proteins also found in smaller, more specialized categories. While these data do not imply a similar gene expression in bladder biopsies of e.g. lymphoma and ulcerative IC patients, the data suggest a relationship between these two diseases, primarily due to the shared importance of B-cell markers.

**Table 5:** Diseases with signature genes similar to the gene expression profile of ulcerative IC bladder biopsies

**#**	**disease**	**p-value**	**ratio**
1	Immune System Diseases	1.02E-35	582/2482
2	Lymphoproliferative Disorders	1.38E-28	382/1498
3	Lymphatic Diseases	1.45E-28	388/1530
4	Immunoproliferative Disorders	1.86E-28	382/1500
5	Hemic and Lymphatic Diseases	2.93E-25	440/1867
6	Autoimmune Diseases	1.85E-23	299/1145
7	Lymphoma	5.93E-21	256/964
8	Leukemia	3.39E-20	280/1098
9	Bacterial Infections and Mycoses	1.36E-19	310/1265
10	Infection	2.47E-19	289/1159
11	Goiter	8.45E-19	59/117
12	Plasmacytoma	6.11E-18	122/367
13	Paraproteinemias	1.38E-17	121/366
14	Hematologic Diseases	1.74E-17	297/1234
15	Sarcoidosis	1.85E-17	56/113
16	Multiple Myeloma	5.93E-17	118/359
17	Hyperthyroidism	7.14E-17	54/109
18	Graves' Disease	9.56E-17	52/103
19	Exophthalmos	9.56E-17	52/103
20	Orbital Diseases	1.63E-16	52/104
21	Joint Diseases	2.37E-16	197/737
22	Blood Protein Disorders	2.38E-16	123/387
23	Leukemia, Lymphocytic	2.95E-16	148/502
24	Arthritis	4.92E-16	190/707
25	Hemorrhagic Disorders	6.28E-16	142/478
26	Lymphoma, Non-Hodgkin	6.64E-16	160/563
27	Diabetes Mellitus, Type 1	1.46E-15	116/364
28	Vascular Hemostatic Disorders	1.89E-15	124/401
29	Arthritis, Rheumatoid	2.18E-15	159/565
30	Connective Tissue Diseases	2.62E-15	286/1217
31	Rheumatic Diseases	3.94E-15	178/661
32	Gastroenteritis	6.14E-15	117/375
33	Lymphoma, B-Cell	9.28E-15	113/359
34	Inflammatory Bowel Diseases	1.21E-14	103/316
35	Crohn Disease	1.57E-14	64/157
36	Lupus Erythematosus, Systemic	1.68E-14	99/300
37	Hepatitis, Autoimmune	6.39E-14	21/25
38	Virus Diseases	9.56E-14	215/871
39	Intestinal Diseases	2.55E-13	312/1402
40	Lymphoma, Low-Grade	4.01E-13	30/49

### Differences in gene expression in ulcer tissue vs. non-ulcer tissue from patients with Hunner's ulcers ("ulcus-vs-ni")

The populations of five ulcer tissues (4, 12, 13, 14, 15) and five non-ulcer tissues (4 ni, 12 ni, 13 ni, 14 ni, 15 ni) were compared by GeneChip expression array analysis ("ulcus-vs-ni"). From a total of 54,613 tested probe sets 30,842 were found to be present (detection p-value < 0.05). The difference in mRNA levels between the two groups was significant for 505 probe sets as determined by the t-test statistics (p-value < 0.01; FDR 57.2%) (see Additional file 9). One hundred and fourteen of the 505 significant probe sets showed an ulcer vs. non-ulcer expression ratio greater than two (see Additional file 9).

The significantly up-regulated genes of the ulcus-vs-ni comparison had the best overlaps with the GO categories "wound healing" (GO:0042060) for "biological process" (corrected p-value 0.04228), "glycosaminoglycan binding" (GO:0005539) for "molecular function" (corrected p-value 0.06007) and "extracellular region" (GO:0005576) for "cellular component" (corrected p-value 1.35E-08) (ermine J analysis [35]).

## Discussion

Our data provide a global picture of the differences between mRNA levels of bladder biopsies from classic IC patients and from healthy controls. In the ni-vs-healthy comparison, non-ulcer tissue of classic IC patients (ni) and tissue from healthy controls (healthy) have been compared, and in the ulcus-vs-ni comparison, ulcer (ulcus) and non-ulcer tissues (ni) have been compared.

The ni-vs-healthy comparison yielded over 3,500 significant changes at an FDR of 8.7%, which means there is a possibility that a discrete gene could be a false positive. However, the majority of the detected changes can be attributed to genuine differences in non-ulcer and healthy tissue (see Additional file 2). In contrast, the ulcus-vs-ni comparison yielded only 500 significant changes at an FDR of 57.2%, meaning the likelihood is only about 50% that a randomly picked "significant" gene is truly differentially expressed in ulcer vs. non-ulcer tissue (see Additional file 9). This suggests that the ulcer and non-ulcer tissue of patients with Hunner's ulcer have only small differences in gene expression. An analysis with a significantly larger set of patients would probably have the statistical power to identify those changes more reliably. Given our limited set of patients, we obtained a rather unreliable set of approximately 500 differentially expressed genes, and therefore, our results of the associated GO categories should be regarded as an indication of how ulcer and non-ulcer biopsies may differ.

As the gene expression arrays used in our study cover all known human mRNAs (over 47,000 transcripts), previous gene expression data on IC can be compared with our results.

Keay and others have published microarray analysis data investigating gene expression differences in explanted epithelial cells from bladder biopsies [15]. They focused on approximately 4,000 genes and compared differences between IC patients (NIDDK criteria) and asymptomatic controls, and differences between normal epithelial cells treated with APF, or with mock APF. One of the 13 genes discussed in their publication was significant in our ni-vs-healthy comparison. This was "neutral amino acid transporter B" which was, as in their results, down-regulated (see Additional file 4). We anticipated only minor overlaps between the data from their paper and our results since histopathological data from our investigated bladder biopsies indicated that urothelial cells contribute only a minor part of all cell types (Figure 4). Even if there were differences in urothelial gene expression between our two investigated subgroups, they might be too subtle to be detected. Furthermore, many of the 13 genes were ubiquitously expressed genes (e.g. cyclin D, stress-activated protein kinase JNK1, putative tRNA synthetase-like protein, ribosomal protein L27a), and as we primarily examined cell types other than epithelial cells, this must lead to different results.

Hurst and co-workers addressed the question of abnormalities in the urothelium of IC patients [42,43]. With a set of immunostainings against proteoglycan core proteins and differentiation markers, they investigated biopsies of 27 IC patients (NIDDK criteria) and five controls having stress incontinence [42]. They scored their data from -2 (most abnormal) to +2 (normal), and their findings suggested abnormal differentiation in the IC urothelium. Many of the proteins investigated in their study were present in both IC and control biopsies, but were localized at different sites (e.g. dense luminal staining vs. uniform distribution). Gene expression analyses of entire biopsies, as performed in our study, give no information on spatial distributions. Moreover, the amount of urothelium was reduced in our biopsies, and the presence of a protein and its respective mRNA will not necessarily need to correlate. Taking these three points into account, it is expected that genes encoding the proteins found by Hurst would not be significantly regulated in our analysis. Interestingly, two genes, *keratin-20 *and *uroplakin-1b *were considerably up-regulated (20- and 11-fold, respectively) in the healthy controls, in agreement with the findings of Hauser et al. (keratin-20 or uroplakin staining in normal urothelium, no keratin-20 or weak uroplakin staining in most abnormal urothelium) [42].

Generally, the presence of mRNA and the presence of the encoded protein do not need to correlate. Temporal and spatial differences in expression are likely. Regulation of mRNA translation, protein processing, protein stability and protein translocation are all factors that influence a direct correlation between an mRNA and its mature protein.

Inflammatory features in IC have been investigated by histological analysis. Peeker and Fall [4,5] defined a clear picture of ulcerative or classic IC that included urothelial spongiosis and detachment; subepithelial, perineural and perivascular deposits of mononuclear cells; and a characteristic mast cell response, with an increase of mast cells in detrusor muscle and in the lamina propria. In addition, other groups reported extensive bladder inflammation of patients with ulcer [7,20]. With our restriction to classic IC we were able to characterize a relatively homogenous group of patients. While our data agreed with reported histological findings, they additionally provided us with a series of potential gene expression markers for this subtype of the disease.

The generation of a gene expression profile for non-ulcerative IC is expected to be more difficult. In contrast to classic IC, literature regarding non-ulcerative IC described a heterogeneous picture with respect to inflammation. Peeker and Fall [4] selected their patients according to the NIDDK criteria and reported no inflammatory signs and a less prominent mast cell involvement in non-ulcerative IC. Erickson et al. [44] also evaluated their patients according to the NIDDK cystoscopic criteria. Severe inflammation was seen in 30% of the patients who met the criteria and in 23% who did not. Ulcers were reported for only four patients (corresponding to 11% of the patients who met the criteria). Also Denson et al. [21], who used the NIDDK inclusion criteria, described mononuclear infiltration as the most consistent histological feature (while only 1% of their patients had a Hunner's ulcer, 70% showed mild to severe inflammation). In another study by Erickson et al. [7], up to 35% of the non-ulcerative patients had severe inflammations.

We can conclude that patient evaluation and selection for a gene expression profile of non-ulcerative IC is crucial and challenging. Very likely, a careful subdivision of this heterogeneous patient group will be indispensable to obtain statistically significant results.

An interesting and unexpected result of our study was the severe inflammatory infiltration at non-ulcer ("ni") sites of a bladder with Hunner's ulcer (Figure 3, Additional file 3). In the literature, many research groups have not focused on such tissue, but have instead analyzed biopsies of the most severely affected part of the bladder (e.g. [7,20,21]). Factors released from an ulcer region, or, more generally, from an abnormal urothelium, may have paracrine influences on surrounding cells. For example, TNF alpha (6.5-fold overexpressed in our ni-vs-healthy comparison) plays a central role in pro-inflammatory paracrine signaling and mediates the recruitment of cells involved in inflammatory and wound healing responses [45]. It was not clear to us why gene expression of biopsy 14 ni was similar to the healthy controls (Figure 1). A potential reason might be a reduced paracrine signaling at the location of biopsy.

Compared to controls, mast cell counts were 6- to 8-fold higher in the detrusor muscle of classic IC, and 2- to 3-fold higher in non-ulcerative IC [9-11].

Several reviews attribute a key role to mast cells in the mediation of chronic inflammation. Signals released from abnormal urothelium or penetrating through a damaged urothelium may stimulate submucosal sensory nerves, and recruit and activate inflammatory leukocytes including mast cells. Upon activation, mast cells release preformed mediators stored in granules (histamine, heparin, neutral proteases), generate mediators by enzymatic processing of membrane phospholipids (leukotrienes, prostaglandins), and produce cytokines and chemokines de novo (IL-6, IL-8, TNF and many others). Released mediators initiate and maintain the recruitment of inflammatory cells, promote angiogenesis and stimulate fibrosis [9,46-48].

Upon histological analysis, mastocytosis in the deeper areas of the bladder wall was found in all of our classic IC patients. The concept outlined above describing the central role of mast cells in inflammation may explain many of our findings. Upon cystoscopy and bladder filling, the bladder wall was found to be very fragile at many points, not only at the sites of Hunner's ulcers (unpublished observation).

## Conclusion

GeneChip expression arrays with total-RNA isolated from bladder biopsies provided molecular insights into ulcerative IC. The comparison of IC patients with healthy controls revealed approximately 1,000 genes with an IC-to-healthy expression ratio greater than two. Most differences in gene expression are not due to induction or repression of individual genes, as is observed by stimulation of cell cultures in vitro, but are rather a consequence of leukocyte infiltration. The predominant process of ulcerative IC is an extensive immune response. B-cell commitment and the production of antibodies appear to be striking features, and most likely distinguish this chronic sterile cystitis from an acute infectious cystitis. Previous histopathology data have shown that leukocyte infiltration was common in ulcerative IC. Our data clearly confirm these findings, and, additionally, provide us with a pattern of potential molecular markers characterizing this subtype of IC. Future studies analyzing bladder biopsies of patients with similar symptoms, but not having ulcerative IC, will be needed. This would include the comparison of our ulcerative IC data with data from patients having non-ulcerative IC, urinary tract infection, bladder cancer, urinary tract stones or chemical/radiation cystitis. The assemblage and evaluation of such data would provide us with molecular information for a differential diagnosis of IC and other bladder diseases.

## Methods

### Patients and tissue samples

IC patients were evaluated according to the NIDDK criteria. Before surgery, urine was collected by urinary catheterization. Urine specimens were inoculated for bacterial cultures and tested for bacterial growth. Standard clinical and laboratory diagnostic procedures were performed. Informed consent was obtained from all patients and a self-completed questionnaire was filled out. Cystoscopy and surgery were performed under general anesthesia. Three to four cold-cup biopsies (20 mm^3 ^size) from bladder tissue excluding the trigone were collected. Immediately after biopsy removal the bleeding was stopped by heat-coagulation. Clinical research has been approved by the ethics commission (Ethikkommission des Kantons Thurgau, Switzerland) and was in compliance with the Helsinki Declaration.

### Tissue storage and RNA isolation

At surgery, biopsies were submerged in 1 ml RNAlater (76104, QIAGEN, Valencia, CA, USA) and stored according to the manufacturer. For RNA isolation, biopsies were transferred to 600 μl RLT buffer (RNeasy Mini Kit 74104, QIAGEN, Valencia, CA, USA) containing 143 mM β-mercaptoethanol and homogenized with the Dispomix system (Medic Tools, Zug, Switzerland) using mechanical disruption conditions of 4 × 15 sec at 4000 rpm. After incubation at room temperature for 15 minutes and centrifugation at 10,000 × g for 3 minutes, the supernatant was treated according to the RNeasy Mini Kit protocol. An on-column DNaseI digestion (79254, QIAGEN, Valencia, CA, USA) was performed following the handbook for the RNeasy Mini Kit. The quality of the isolated RNA was determined with a NanoDrop ND 1000 (NanoDrop Technologies, Delaware, USA) and a Bioanalyzer 2100 (Agilent, Waldbronn, Germany). Only those samples with a 260 nm/280 nm ratio between 1.8 – 2.1 and a 28S/18S ratio within 1.5 – 2 were processed further.

### cRNA preparation

RNA samples (1 μg) were reverse-transcribed into double-stranded cDNA with One-Cycle cDNA Synthesis Kit (Affymetrix Inc., P/N 900431, Santa Clara, CA). The double-stranded cDNA was purified using a Sample Cleanup Module (Affymetrix Inc., P/N 900371, Santa Clara, CA). The purified double-stranded cDNA was in vitro transcribed in the presence of biotin-labeled nucleotides using an IVT Labeling Kit (Affymetrix Inc., P/N 900449, Santa Clara, CA). The biotinylated cRNA was purified using a Sample Cleanup Module (Affymetrix Inc., P/N 900371, Santa Clara, CA) and its quality and quantity was determined using NanoDrop ND 1000 and Bioanalyzer 2100.

### Array hybridization

Biotin-labeled cRNA samples (13 μg) were fragmented randomly to 35–200 bp at 94°C in Fragmentation Buffer (Affymetrix Inc., P/N 900371, Santa Clara, CA) and were mixed in 300 μl of hybridization buffer containing a hybridization Control cRNA and Control Oligo B2 (Affymetrix Inc., P/N 900454, Santa Clara, CA), 0.1 mg/ml herring sperm DNA and 0.5 mg/ml acetylated bovine serum albumin in 2-(4-morpholino)-ethane sulfonic acid (MES) buffer, pH 6.7, before hybridization to Affymetrix-GeneChip^® ^Human Genome U133 Plus 2.0 arrays for 16 h at 45°C. Arrays were then washed using an Affymetrix Fluidics Station 450 FS450_0001 protocol. An Affymetrix GeneChip Scanner 3000 (Affymetrix Inc., Santa Clara, CA) was used to measure the fluorescent intensity emitted by the labeled target.

### Statistical analysis

Raw data processing was performed using the Affymetrix GCOS 1.4 software (Affymetrix Inc., Santa Clara, CA). After hybridization and scanning, probe cell intensities were calculated and summarized for the respective probe sets by means of the MAS5 algorithm [49]. To compare the expression values of the genes from chip to chip, global scaling was performed, which resulted in the normalization of the trimmed mean of each chip to a target intensity (TGT value) of 500 as detailed in the statistical algorithms description document from Affymetrix [50]. Quality control measures were considered before performing the statistical analysis. These included adequate scaling factors (between 1 and 3 for all samples) and appropriate numbers of present calls calculated by application of a signed-rank call algorithm [51]. The efficiency of the labeling reaction and the hybridization performance were controlled using the following parameters: Present calls and optimal 3'/5' hybridization ratios (around 1) for the housekeeping genes (GAPDH and ACO7), for the poly-A spike in controls, and the prokaryotic control (BIOB, BIOC, CREX, BIODN).

### Data processing and further analysis

A probe set was considered as expressed ("present") in a sample if the corresponding detection p-value was below 0.05. For further normalization and analysis the expression signals were transformed to log-scale. The log-expression values were normalized such that the average of expression of those genes expressed in all samples was identical. Differential expression was assessed for those probe sets that were considered present in at least four samples. By this we discarded the "absent" probe sets that would unnecessarily inflate the number of false positives. A probe set was considered significantly differentially expressed if the p-value computed by Student's t-test on the log-intensity data was below 0.01. Gene ontology (GO) analysis was done with the ermineJ software program [35] where we used the standard overrepresentation analysis that is based on Fisher's Exact test. We considered the significantly up- and down-regulated genes separately and searched for the GO categories where these genes are significantly overrepresented. Pathways, processes and disease analyses of significantly regulated genes were performed using MetaCore (GeneGo, St. Joseph, MI, USA).

### Accession number

The Affymetrix microarray related data were submitted to Gene Expression Omnibus (GEO) under accession number: [GSE11783].

### Histopathology data of urinary bladder tissue

At surgery, biopsies of approximately 20 mm^3 ^in size were submerged in buffered formalin. After the dehydration procedure the tissue was embedded in paraffin. At least three serial sections of 5 to 7 μm thickness were then cut with a horizontal microtome. The slides were stained with hematoxylin and eosin (HE) or Giemsa and Elastin van Gieson following standard procedures [52], and were analyzed by the Mirax-System (Carl Zeiss AG, Feldbach, Switzerland).

### DNA isolation and PCR analysis of potential pathogens

DNA isolation from bladder biopsies and subsequent PCR analyses were performed by standard validated techniques at IMD Institute for Medical and Molecular Diagnostics Ltd in Zurich, Switzerland. The following pathogens were tested: Chlamydia trachomatis, Helicobacter pylori, Herpes simplex virus, Varicella/Zoster virus, Borrelia burgdorferi, Ureaplasma urealyticum or Mycoplasma genitalium. Also, PCR amplification of the conserved region of bacterial 16S rRNA genes was performed.

## Abbreviations

IC: Interstitial cystitis; ni: non-ulcer tissue in patients with Hunner's ulcers; ulcus: ulcer tissue; NIDDK: National Institute of Diabetes and Digestive and Kidney Diseases; APF: antiproliferative factor; HB-EGF: heparin-binding epidermal growth factor-like growth factor; EGF: epidermal growth factor; IL: interleukin; TNF: tumor necrosis factor; BCR-pathway: B-cell antigen receptor-pathway; GO: Gene ontology; FDR: false discovery rate; HE stain: hematoxylin and eosin staining.

## Authors' contributions

MG planned, coordinated and performed the research, updated the clinical protocols, analyzed the data and wrote the manuscript. VV coordinated the patient recruitment. VG coordinated the patient recruitment, helped organize the clinical study protocol and phrased the study description for the ethics commission. JE coordinated the patient recruitment and phrased the study description for the ethics commission. JB performed the bladder biopsies. CM was responsible for the histopathology data. HR did the statistical analysis of the gene expression arrays and contributed to the manuscript. RM initiated and supervised the research project, assisted in phrasing the initial study description and in writing the manuscript. All authors read and approved the final manuscript.

## Supplementary Material

Additional file 1 **Matrix of 31,579 present probe sets against six healthy, five ni and five ulcer samples**. Statistical requirement: One probe set needed to be present (detection p-value < 0.05) in at least four of the arrays 4 ni, 12 ni, 13 ni, 14 ni, 15 ni, 2 healthy, 5 healthy, 7 healthy, 9 healthy, 10 healthy, 11 healthy. The numbers give the expression values relative to the average of the healthy controls. The values are not logarithmic. Click here for file

Additional file 2 **Matrix of the 3,618 significant probe sets and the corresponding calculated log2 ratios, p-values and FDRs of the t-test**. Probe sets that are significant between the ni and the healthy groups were defined as having p-values < 0.01, the individual FDRs ranged from 3.5% to 8.7%. The table has been sorted starting with the probe set having the best (the lowest) p-value. Click here for file

Additional file 3 **Matrix of the 3,618 significant probe sets against the the six healthy, five ni and five ulcer samples**. The numbers give the log2 ratio of the expression values of the individual IC samples to the average of the healthy controls. To make a ranking list of all probe sets, the five ni data sets of each probe set were averaged ("Avg log2Ratio ni over healthy"). From this number, the overexpression was calculated. Then, the table was sorted starting with the probe set having the highest overexpression. Probe sets that were more than 2-fold up- or down-regulated have been marked in color. In total, there were 1,957 probe sets. Click here for file

Additional file 4 **Matrix of the 2,543 significant proteins (MetaCore analysis, GeneGo) against five ni samples**. The numbers give the log2 ratio of the expression values of the individual IC samples to the average of the healthy controls. To make a ranking list of all proteins, the five ni data sets of each protein have been averaged ("Avg log2Ratio ni-vs-healthy"). From this number, the overexpression was calculated. Then, the table was sorted starting with the protein having the highest overexpression. Proteins that were more than 2-fold up- or down-regulated have been marked in color. In total, there were 1,417 proteins. Click here for file

Additional file 5 **Matrix of the top 25 proteins (MetaCore analysis, GeneGo) against six healthy, five ni and five ulcer samples**. The numbers give the log2 ratio of the expression values of the individual samples to the average of the healthy controls (similar to Additional file 4). Overexpression was calculated as described (see Additional file 4). The expression of the top 25 proteins is shown. The two "gene symbols" with no assigned proteins (LOC728320 and LOC100133862, see Additional file 4) have been omitted. The calculated overexpression data are also shown in Table 2. Click here for file

Additional file 6 **Matrix of the top 100 B-cell probe sets, the corresponding gene names and the results of the clustering analysis (clustering number and clustering colors)**. Biomarker search with Genevestigator [33]. The algorithm has not yet been published. The target "B-lymphocytes" was compared to all high quality human data, and the top 100 probe sets were determined. Gene expression array results for these probe sets ("presents") were loaded and clustered. Clustering results are shown in Figure 3A. Click here for file

Additional file 7 **Matrix of the top 100 T-cell probe sets, the corresponding gene names and the results of the clustering analysis (clustering number and clustering colors)**. Biomarker search with Genevestigator [33]. The algorithm has not yet been published. The target "T-lymphocytes" was compared to all high quality human data, and the top 100 probe sets were determined. Gene expression array results for these probe sets ("presents") were loaded and clustered. Clustering results are shown in Figure 3B. Click here for file

Additional file 8 **Matrix of the 78 significantly regulated proteins against the calculated overexpression factor**. Top process from the MetaCore analysis with a ratio of 78/206 and a p-value of 3.05E-18. The results of the five ni samples were averaged (not shown), then the overexpression was calculated (shown) and the results were sorted starting with the highest gene expression. Click here for file

Additional file 9 **Matrix of the 505 significant probe sets and the corresponding calculated log2 ratios, p-values and FDRs of the t-test**. Probe sets that were significant between the ulcer and the ni groups were defined as having p-values < 0.01. The individual FDRs ranged from 56.5% to 57.2%. The table was sorted starting with the highest log2 ratio (highest expression ratio ulcus-vs-ni). The 114 probe sets with > 2-fold up- or down-regulation were marked in color. Click here for file
